# Genome-Wide Mutant Screening in Yeast Reveals that the Cell Wall is a First Shield to Discriminate Light From Heavy Lanthanides

**DOI:** 10.3389/fmicb.2022.881535

**Published:** 2022-05-19

**Authors:** Nicolas Grosjean, Marie Le Jean, Michel Chalot, Héctor M. Mora-Montes, Jean Armengaud, Elisabeth M. Gross, Damien Blaudez

**Affiliations:** ^1^Université de Lorraine, CNRS, LIEC, Nancy, France; ^2^Université de Lorraine, CNRS, LIEC, Metz, France; ^3^Laboratoire Chrono-Environnement, Université de Bourgogne Franche-Comté, CNRS, Besançon, France; ^4^Université de Lorraine, Nancy, France; ^5^Departamento de Biología, División de Ciencias Naturales y Exactas, Universidad de Guanajuato, Guanajuato, Mexico; ^6^Département Médicaments et Technologies pour la Santé (DMTS), Université Paris-Saclay, CEA, INRAE, Bagnols-sur-Cèze, France

**Keywords:** lanthanum, ytterbium, cell wall, endocytosis, signaling, deletome

## Abstract

The rapidly expanding utilization of lanthanides (Ln) for the development of new technologies, green energies, and agriculture has raised concerns regarding their impacts on the environment and human health. The absence of characterization of the underlying cellular and molecular mechanisms regarding their toxicity is a caveat in the apprehension of their environmental impacts. We performed genomic phenotyping and molecular physiology analyses of *Saccharomyces cerevisiae* mutants exposed to La and Yb to uncover genes and pathways affecting Ln resistance and toxicity. Ln responses strongly differed from well-known transition metal and from common responses mediated by oxidative compounds. Shared response pathways to La and Yb exposure were associated to lipid metabolism, ion homeostasis, vesicular trafficking, and endocytosis, which represents a putative way of entry for Ln. Cell wall organization and related signaling pathways allowed for the discrimination of light and heavy Ln. Mutants in cell wall integrity-related proteins (e.g., Kre1p, Kre6p) or in the activation of secretory pathway and cell wall proteins (e.g., Kex2p, Kex1p) were resistant to Yb but sensitive to La. Exposure of WT yeast to the serine protease inhibitor tosyl phenylalanyl chloromethyl ketone mimicked the phenotype of *kex2*∆ under Ln, strengthening these results. Our data also suggest that the relative proportions of chitin and phosphomannan could modulate the proportion of functional groups (phosphates and carboxylates) to which La and Yb could differentially bind. Moreover, we showed that *kex2*∆, *kex1*∆, *kre1*∆, and *kre6*∆ strains were all sensitive to light Ln (La to Eu), while being increasingly resistant to heavier Ln. Finally, shotgun proteomic analyses identified modulated proteins in *kex2*∆ exposed to Ln, among which several plasmalemma ion transporters that were less abundant and that could play a role in Yb uptake. By combining these different approaches, we unraveled that cell wall components not only act in Ln adsorption but are also active signal effectors allowing cells to differentiate light and heavy Ln. This work paves the way for future investigations to the better understanding of Ln toxicity in higher eukaryotes.

## Introduction

Lanthanides (Ln) are 15 chemical elements belonging to the rare earth elements (REEs). Two subgroups are usually distinguished based on both the atomic mass and the ionic radius, namely, light REEs (LREEs, La to Eu) and heavy REEs (HREEs, Gd to Lu). These so-called technology-critical elements are key elements in energetic transition, high technology (Hu et al., 2018), medicine, and technological military devices and are used as fertilizers and food supplements in agriculture (He et al., 2001; Hu et al., 2004; Ciacci et al., 2015). High concentrations of lanthanides can be found in soil of mining areas but also have been reported to accumulate in kidney, bone, and brain tissues after exposition to MRI contrasting agent (Ramalho et al., 2016; Gulani et al., 2017). As emerging contaminants, their large production and utilization raise the question of their impact on the environment and human health (Noack et al., 2014; Lee and Wen, 2018).

Although Ln have been considered harmless and non-essential elements (Leeuw and Academic, 2000), results from the last few years reveal the effects of Ln on living organisms. They are cofactors for pyrroloquinoline quinone-dependent methanol/ethanol dehydrogenases for certain bacteria (Nakagawa et al., 2012; Pol et al., 2014; Skovran and Martinez-Gomez, 2015; Good et al., 2016; Wehrmann et al., 2017; Cotruvo, 2019) but also present antibacterial, antifungal, and nematocidal activities (Wakabayashi et al., 2016) by increasing membrane permeability (Weiwei et al., 2007; Yufeng et al., 2007), generate oxidative stress (Pagano et al., 2015), induce mitotic and chromosomal aberrations (Pagano et al., 2015), and impair human health (Wei et al., 2013; Noack et al., 2014; Gulani et al., 2017). However, the few existing studies mentioning toxicity remain elusive, which reflects the lack of data on cellular and molecular mechanisms causing Ln toxicity.

A few studies have hypothesized that LREEs and HREEs may induce different responses, based on their relative reactivity and chemical differences (Gonzalez et al., 2014). In addition to their different toxicity levels (HREEs > LREEs (Tyler, 2004; Gonzalez et al., 2014; Goecke et al., 2017)), only a few studies revealed the specific modes of action of these two REE groups with a single multi-scale analysis directly comparing the differential effect of two Ln (Grosjean et al., 2021). As such, specific accumulation of LREEs has been reported in ferns (Grosjean et al., 2020), while angiosperms preferentially translocate HREEs in their aboveground tissues (Liu et al., 2018; Grosjean et al., 2019), thereby suggesting different transport systems between LREEs and HREEs. Molecular evidence also supports this hypothesis in S*accharomyces cerevisiae*, in which disruption of the Ca channel Cch1p/Mid1p restricts La but not Gd uptake (Ene et al., 2015). Therefore, it is essential to unravel the distinct molecular and cellular effects of LREEs and HREEs, since diverse exposure scenarios may occur in the environment, with potentially vastly different consequences for the biota.

The screening of a mutant collection of *S. cerevisiae* has previously proven to be a powerful approach to pinpoint the role of non-essential proteins in toxicity modulation when exposed to different environmental stressors, such as metals (Scherens and Goffeau, 2004; Jin et al., 2008). Based on the phylogenetic relationships between *S. cerevisiae* and other eukaryotes and the strong conservation of basic stress responses, the mechanisms revealed through this approach shed light on pathways that may also be involved in other eukaryotes (Botstein et al., 1997; Kachroo et al., 2017). Recently, a pooled population of yeast mutants was screened with low Ln concentrations revealing a limited set of mutants and pathways (Pallares et al., 2021). However, while the use of pooled population for mutant screening allows the high-throughput generation of data, it also introduces several biases. For instance, mutant cells can affect one another confounding the results of a pooled screen, generating false positives (So et al., 2019), or cell interactions and competition masking minor loss-of-function phenotype (Kim et al., 2018). Therefore, complementary screening methods are needed to grasp the full response profile of *S. cerevisiae* to an acute Ln exposure. For this purpose, we leveraged an arrayed genome-wide phenotyping screen of the entire set of gene deletion mutants to identify gene products that modulate Ln cellular toxicity at high concentrations of Ln. By employing a method vastly used for other toxicants (heavy metals, chemicals, and physical stressors), it provides an opportunity to compare Ln cellular impacts to other stressors. In this regard, we hypothesized that Ln would present distinct effects from known toxicants and that LREEs and HREEs would induce specific patterns. Therefore, given their relative environmental abundances and utilizations among lanthanides, we selected La and Yb as their respective representatives. The genome-wide mutant screen allowed us to pinpoint the cellular compartments and functions affected by Ln, such as the cell wall organization that we further investigated because of a distinct response between La and Yb. Numerous genes/functions impacted by Ln are conserved in humans and provide new promising hypotheses to study Ln-mediated toxicity in humans.

## Materials and Methods

### Yeast Strains and Chemicals

Deletion mutants as well as the wild-type *Saccharomyces cerevisiae* strains BY4741 (MATa; *his3*Δ1; *leu2*Δ0; *met15*Δ0; *ura3*Δ0) and BY4742 (MATα; *his3*Δ1; *leu2*Δ0; *lys2*Δ0; *ura3*Δ0), both isogenic to the S288C strain, were used in this study. They were purchased from EUROSCARF. The haploid deletion library consists of 4,733 mutants for non-essential genes. All Ln were purchased from Sigma-Aldrich (MO, USA) as hydrated chloride salts (LnCl_3_, xH_2_O). The Kex2 gene was synthesized and cloned into the pYES2 plasmid at the NotI restriction site to obtain the expression vector pYES2-*KEX2*, under the activity of the *GAL1* promoter.

### *Saccharomyces cerevisiae* Genomic Phenotypic Screen With Lanthanides

The 4,733 mutants (BY4741 background) were used for genomic phenotyping. Two Ln (La and Yb) were selected and used as chloride forms (LaCl_3_ and YbCl_3_, respectively). Ln concentrations for the screen were defined by pilot experiments as 4.5 mM La and 3.8 mM Yb, allowing discrimination in a single step of both sensitive or resistant mutants (Supplementary Figure S1). Briefly, individual deleted mutants were grown in 96-well master plates in 200 μl of YPD (10 g yeast extract, 20 g peptone, 20 g dextrose) at 28°C until the stationary phase. A Thermo Scientific^TM^ Nunc^TM^ Replication System (250520) was used to replica inoculate the surface of YPD agar plates supplemented with either La, Yb, or Ln-free. Plates were digitally recorded after 5 days of growth at 28°C. Ln-sensitive and Ln-resistant mutants were identified when colony size under Ln exposure was decreased or increased, respectively, compared to WT and neighboring mutants but also to the size of colonies on control plates lacking Ln. Four replications were carried out for each condition. YPD medium is commonly used in metal toxicity screen experiments in yeast. However, its high concentration in phosphates promotes the precipitation of Ln/phosphate complexes that are no longer toxic to the cells and thus justify the use of high concentrations of Ln to achieve Ln toxicity. To verify whether the relatively high concentrations of La and Yb used in this experiment had an influence on the quality of the results, a preliminary screen was performed using 350 mutants (randomly selected) on low concentrations of La (300 μM) and Yb (120 μM) in YNB agar medium devoid of inorganic phosphates and supplemented with 1 mM β-glycerophosphate. No Ln precipitation was visible in this medium. Identical results (26 sensitive or resistant mutants) were obtained for all 350 mutants between the YPD and YNB screening experiments, refuting a putative bias with high Ln concentrations in the YPD medium. Additionally, some mutants grew poorly on YNB medium making screening results more difficult to assess. For these reasons, YPD agar medium was used for the entire primary screen, as well as for the validation screen.

### Genomic Phenotypic Validation Screen

Mutants identified as Ln-sensitive or Ln-resistant in the primary screen were individually confirmed by serial dilution spot assays. Mutants were grown as previously described, and serial tenfold increment dilutions were performed. Five microliters of six dilutions were spotted on YPD agar plates containing 4.5 mM La or 3.8 mM Yb to verify Ln-sensitive mutants and 4.72 mM La and 4 mM Yb for Ln-resistant mutants. The results were observed after 5 days of growth at 28°C. Sensitivity and resistance levels were assigned to the mutants according to the number of dilutions where cells grew. Consequently, mutants exhibiting a reduction in colony-forming ability at the first, second-third, or fourth-fifth dilutions were classified as “high” (HS), “medium” (MS), or “low” (LS) sensitive, respectively. Conversely, mutant strains exhibiting an increase in colony-forming ability at the first-second, third-fourth, or fifth-sixth dilutions were classified as “low” (LR), “medium” (MR), or “high” (HR) resistance, respectively. Twenty-five mutants randomly picked in the identified mutants were also assayed in the haploid MATα strain BY4742 to validate the robustness of the screen.

### Serine Protease Inhibition Assay

To investigate the effect of the serine protease inhibitor tosyl phenylalanyl chloromethyl ketone (TPCK) on Ln resistance, drop tests of 3-fold serial dilutions of the wild-type strain were exposed to La (3.8 mM) and Yb (3.2 mM) with the addition of 80 μM TPCK in solid YPD and grown for 5 days at 28°C.

#### Chitin Staining

Wild-type and mutant cells were grown in YNB medium as described above. Cultures were inoculated at an OD_600nm_ of 0.05 and grown overnight, with or without the addition of 160 μM La or 8 μM Yb, concentrations corresponding to the EC_50_ of the wild-type strain. Chitin was stained as described by Okada et al. (2010). Briefly, yeast cells were harvested at 4000 rpm, rinsed twice with deionized water, and stained with 1 mg/ml calcofluor white (Sigma-Aldrich) for 1 min. Stained cells were washed once with deionized water and resuspended in water to be visualized by fluorescence microscopy using a DAPI filter (excitation band: 300–400 nm; emission band >420 nm).

### Compositional Analysis of the Cell Wall

Cells were mechanically prepared in a Braun homogenizer and processed as described elsewhere (Mora-Montes et al., 2007). Briefly, cell homogenates were centrifuged for 10 min at 20000 x g and 4°C, and the pellet was saved and washed five times with deionized water. Then, the cell walls were cleansed by serial incubations with hot SDS, β-mercaptoethanol, and NaCl and then hydrolyzed with 2 M trifluoroacetic acid (Sigma-Aldrich). Aliquots of 20 μl of acid-hydrolyzed samples were analyzed by high-performance anion exchange chromatography with pulsed amperometric detection in a Dionex system (Thermo Fisher Scientific) using separation conditions reported elsewhere (Estrada-Mata et al., 2016). To determine the protein concentration, cleansed walls were alkali-hydrolyzed as reported (Mora-Montes et al., 2007) before quantification using the Pierce BCA Protein Assay (Thermo Fisher Scientific).

The phosphomannan content was inferred from the cell’s ability to bind the Alcian blue dye (Hobson et al., 2004). Upon growth, the cell concentration was adjusted to an OD_600 nm_ of 0.2 in deionized water, and 1 ml aliquots were used to interact with 30 μg/ml Alcian blue (Sigma-Aldrich) and analyzed as described (Hobson et al., 2004). The cell wall mannan content was quantified essentially as previously reported (Navarro-Arias et al., 2016). For *O*-linked or *N*-linked mannan trimming, cells were incubated with 1 N NaOH and gently shaken for 18 h at room temperature or for 20 h at 37°C with 25 U endoglycosidase H (New England Biolabs). In both cases, cells were pelleted by centrifugation, and the supernatants were collected, lyophilized, and analyzed by high-performance anion exchange chromatography with pulsed amperometric detection (Mora-Montes et al., 2012).

### Determination of Lanthanide Contents in Yeast by Inductively Coupled Plasma Mass Spectrometry

Mutant and wild-type (BY4741) strains were grown in liquid YNB medium lacking inorganic phosphates and supplemented with β-glycerophosphate (1 mM). Cultures were inoculated at an OD_600nm_ of 0.05 (160 rpm; 28°C), and once the cultures reached an OD_600nm_ of 0.8, Ln were added at 50 μM and 6 μM La and Yb, respectively, concentrations corresponding to the EC_10_ of the wild-type strain, and were exposed for 1 h. Cells were harvested at 4000 rpm for 1 min at 4°C. Pellets were washed three times with ice-cold MES (20 mM)—EDTA (10 mM) buffer (pH 6) followed by three washings with ice-cold ultrapure water and dried for 48 h at 70°C. Triplicate samples were prepared. Elemental extraction was performed on aliquots (125 mg) of each sample *via* mineralization with 1.75 ml HNO_3_ and 0.5 ml hydrogen peroxide in closed tubes placed in a block digestion system (DigiPREP, SCP Sciences, Courtaboeuf, France). A gradual heating mode was used to achieve a final temperature of 100°C (total run of 265 min). Finally, ultrapure water was added to 12.5 ml, and filtration to 1 μm was performed. Concentrations were determined in triplicate using inductively coupled plasma mass spectrometry (ICP-MS, X Series II Model, Thermo Fischer Scientific, Courtaboeuf, France). The validity of the analytical method was checked by means of standard reference material (Oriental basma tobacco leaves, INCT-OBTL-5, LGC Promochem, Molsheim, France).

### Proteomics Analysis

The materials and procedures used for sample preparation for proteomics analysis, tandem mass spectrometry and peptide-to-spectrum assignment and protein identification are fully described in Appendix A.

### Data Analysis

Enriched biological pathways in Ln-stressed conditions vs. control conditions were identified using GSEA software v2.0 (Mootha et al., 2003; Subramanian et al., 2005). To establish a gene ranking, the sensitivity value difference was used. *p*-values for enriched gene sets were defined by computing 1,000 gene set permutations. *Saccharomyces cerevisiae* GO (biological process) derived from the MSigDB format gene set list was downloaded from the GO2Msig database and used as a template. From the GO2Msig datasets, gene set sizes were restricted to a maximum of 300 and a minimum of 10. GSEA results were visualized using Cytoscape v3.4 software (Shannon et al., 2003) and the Enrichment Map v2.1.0 (Merico et al., 2010) plug-in with default settings. In computed enrichment maps, node color corresponds to sensitivity of the mutant to Ln stress (yellow for resistant and blue for sensitive), and edge thickness represents redundancies between two gene sets.

Putative human homolog(s) of yeast gene(s) and any of their associated OMIM disease phenotypes were retrieved from the SGD YeastMine platform.^1^ Biological process and cellular compartment (MIPS cc) analyses were performed using FunSpec^2^ and evaluated for statistical significance (cutoff: value of *p*<0.01). Clustering (heatmaps) was performed using the R package pheatmap. Ln-responsive mutants found in this study were compared with those from other studies carried out on *Saccharomyces cerevisiae* deletion mutant collections on Y (Grosjean et al., 2018), Cd (Jin et al., 2008; Ruotolo et al., 2008; Serero et al., 2008; Thorsen et al., 2009), Ni (Ruotolo et al., 2008; Arita et al., 2009; Bleackley et al., 2011), Zn (Pagani et al., 2007; Wang et al., 2007; Jin et al., 2008; Bleackley et al., 2011), Al (Kakimoto et al., 2005; Tun et al., 2014), As (Haugen et al., 2004; Thorsen et al., 2006, 2009; Jin et al., 2008; Johnson et al., 2016a), Cu (Wang et al., 2007; Jin et al., 2008; Jo et al., 2008; Bleackley et al., 2011), Fe (Wang et al., 2007; Jo et al., 2008; Bleackley et al., 2011), Mn (Wang et al., 2007; Bleackley et al., 2011; Chesi et al., 2012), Co (Bleackley et al., 2011), or Cr (Jin et al., 2008; Johnson et al., 2016b) and other stressors (Bennett et al., 2001; Chang et al., 2002; Aouida et al., 2004; Serrano et al., 2004; Thorpe et al., 2004).

### Statistical Analyses

For statistical analyses, one-way ANOVA and Tukey HSD post-hoc tests were used. Two-tailed Student’s *t*-test was used where appropriate. All analyses were performed using R (version 3.4.1). All reported values are the means ± standard deviation (SD) of triplicates (*n* = 3).

### Data Availability

The authors declare that all data related to the findings of this study are available within the article and supplementary information or are available from the corresponding author upon reasonable request. Source data are provided with this paper. The mass spectrometry proteomics data for the *kex2*∆ mutant have been deposited to the ProteomeXchange Consortium *via* the PRIDE partner repository with the dataset identifier PXD025030 and project DOI 10.6019/PXD025030.^3^ The mass spectrometry and proteomics dataset for the WT are available under the dataset identifier PXD010700 and project DOI 10.6019/PXD010700.

## Results and Discussion

### Yeast Genomic Phenotyping Unravels a Large Set of Lanthanide-Responsive Genes

The complete set of 4,733 haploid non-essential gene deletion mutants (BY4741, MATa) was screened under sublethal La and Yb concentrations. Mutants displaying a resistant or sensitive phenotype were further confirmed. Wild-type sublethal concentrations were used to confirm sensitive mutants, while a wild-type lethal concentrations were used to confirm resistant mutants. The primary and validation screens revealed genes involved either in resistance or sensitivity to Ln based on the modified growth of the mutants toward one or both elements. They were classified into six different categories, ranging from high sensitivity (HS) to high resistance (HR; Supplementary Table S1). The validity and robustness of the screen were confirmed in the opposite mating type (BY4742, MATα) using 25 mutants randomly selected among sensitive and resistant mutants (Supplementary Table S2). A total of 630 genes were involved in the response of yeast cells to Ln, with 1.5 times more resistant (366) than sensitive mutants (236) to at least one Ln. Three times less mutants were identified in a recent fitness analysis of a pooled mutant population performed with the diploid BY4743 strain (Pallares et al., 2021). This lower number of mutants certainly arose from the fact that low concentrations (EC20) of La and Yb were used suggesting that more toxic concentrations is more appropriate to reveal the entire set of genes involved in the tolerance to Ln. The sublethal/lethal concentrations used here reproduce conditions usually applied for metal toxicogenomics in yeast, resulting in a similar number of sensitive (Ruotolo et al., 2008) and resistant (Ruotolo et al., 2008; Grosjean et al., 2018) mutants.

### La and Yb-Responsive Genes Are Mostly Dissimilar

**Figure 1:**
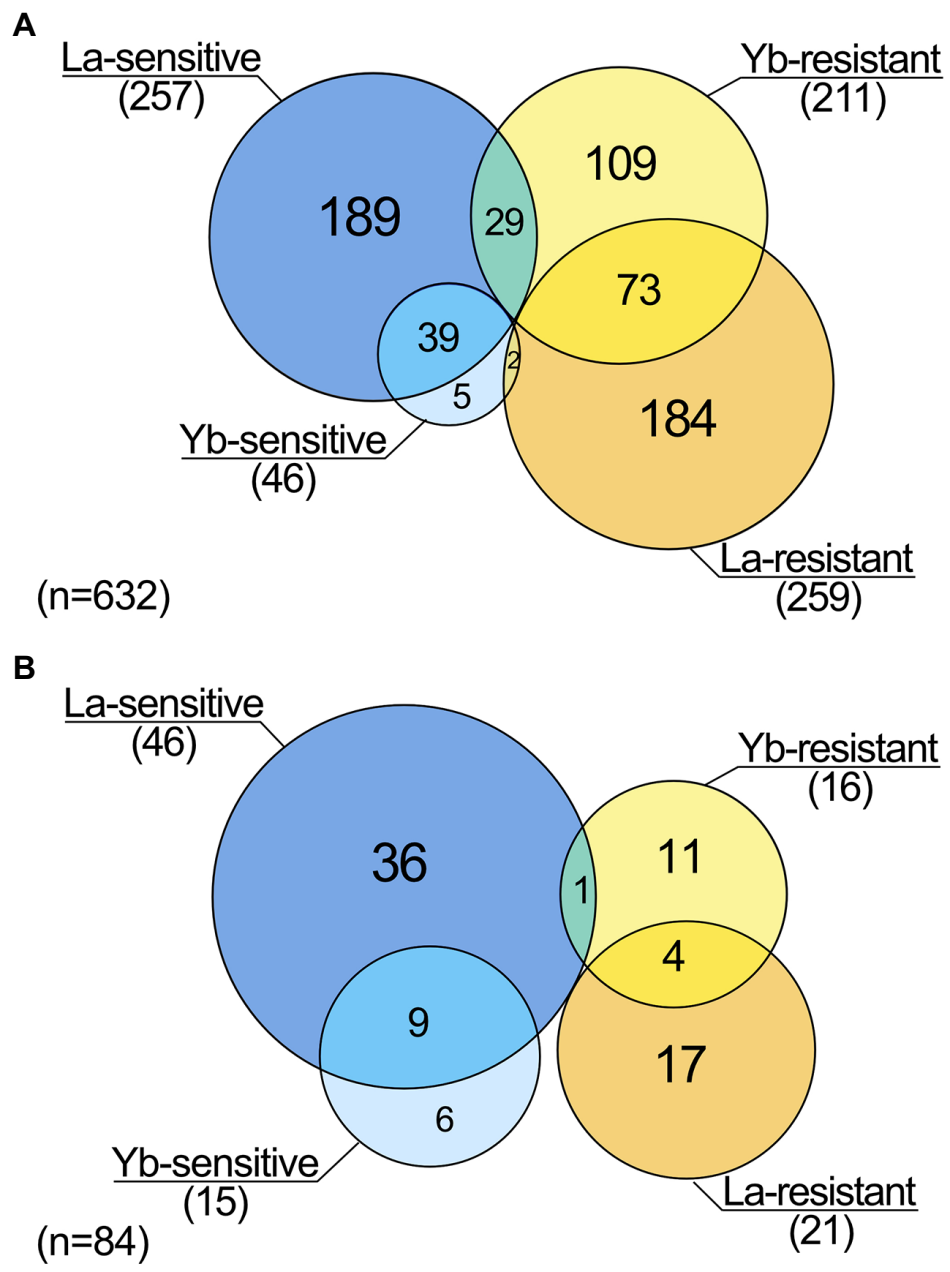
Lanthanide response patterns obtained from genomic phenotyping of a whole mutant collection of *Saccharomyces cerevisiae*. **(A)** Venn diagram highlighting the number of mutants being sensitive or resistant to La and/or to Yb. **(B)** Venn diagram highlighting the number of mutants displaying an exacerbated phenotype (highly resistant/sensitive) toward La and/or Yb.

We identified 259 and 211 genes whose deletion caused resistance to La and Yb, respectively (Figure 1A). Five times more mutants were sensitive to La (257) than to Yb (46), suggesting additional resistance mechanisms toward La. A relatively small proportion of mutants were sensitive (13%, 39 mutants) or resistant (16%, 73 mutants) to both Ln, which reveals the existence of a core, yet minor, set of genes involved in a general Ln response. This core set of mutants is more important than the one identified when using EC20 concentrations of La and Yb (<2%) for the library screen (Pallares et al., 2021). However, in our study, a set of mutants exhibited an opposite phenotype to the two Ln, with 26 mutants being La-sensitive and Yb-resistant, while two displayed a La-resistant and Yb-sensitive phenotype (Figure 1A). Similar proportions were found when considering only the mutants displaying the strongest resistant/sensitive phenotypes (Figure 1B; Supplementary Table S3). Altogether, our data strongly highlight the dissimilarity of responses toward La and Yb in yeast.

### Yeast Lanthanide-Responsive Genes Have Counterparts Whose Mutation Trigger Human Diseases

Human homologues and putative-related diseases were retrieved from the YeastMine database for the set of mutants identified. At least one human homolog was found for 62% (391 mutants) of the knockout genes in mutants resistant or sensitive to Ln (Supplementary Table S1), a third of which are involved in cancers and other human diseases. Ln toxicity concerns are growing after several studies reporting Gd accumulation in bone tissues and more recently in the brain (Ramalho et al., 2016; Gulani et al., 2017). Therefore, the modified fitness toward Ln toxicity observed in mutants for genes associated with bone mineral deficiencies (*SAC6*) or associated with cerebral diseases (e.g., *ELO3*, *PHO85*) emphasizes that more attention should be given to Ln toxicity. The high number of yeast Ln-responsive mutants for genes holding human counterparts sets a promising basis for further studies on the impact of Ln toxicity on human health.

### Lanthanide Responses Strongly Differ From Well-Known Transition Metal-Mediated Responses

**Figure 2:**
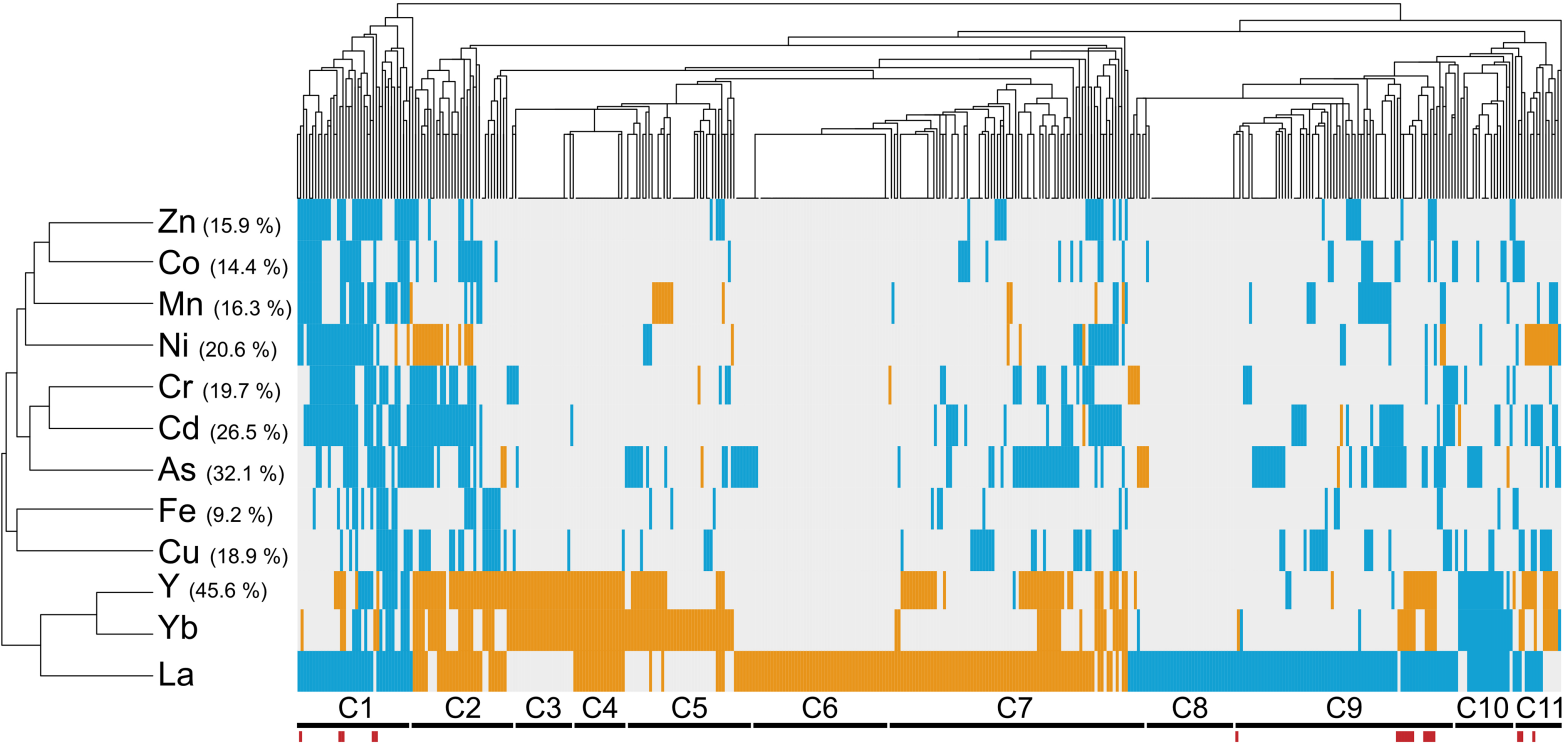
Cross-comparison of mutant phenotypes to lanthanides versus other metallic stressors. Hierarchical clustering of lanthanide sensitivity or resistance-conferring mutations with the mutant response profiles obtained for other metallic stressors. References to these studies are provided in Supplementary Table S4. The x-axis corresponds to gene-deleted mutants, and the y-axis indicates the different stressors from previously published genomic phenotyping screens conducted on yeast deletion mutant collections. Mutants exhibiting either an enhanced sensitivity or resistance compared to the WT are shown in blue and orange, respectively. Correlation was used as distance measurement to cluster mutants based on metal susceptibility. Clusters of mutants are mentioned below (C1–C11), with mutants displaying an opposite phenotype between La and Yb exposure indicated by red bars. Values in brackets denote the percentage of mutants that were found common between the present screen (La and Yb) and screens with other elements.

We performed an *in silico* cross-comparison of Ln-responsive mutants with similar studies carried out with other metals and the metalloid As (Figure 2; Supplementary Table S4). Mutants were grouped into eleven clusters (C1–C11) based on their phenotypes, distinguishing three major patterns.

The first pattern groups REE-specific responsive mutants with 32% mutants that responded specifically to La (17% sensitive (C6) or resistant (C8)), to both Yb and Y (4%, C3), or to La, Yb, and Y (5%, C4). These mutations target genes involved in pathways, such as the response to pheromone-related GO biological processes (*STE* gene family) and chromatin silencing mutants. Twelve mutants (2%, C5) specific to Yb were involved in cell wall biogenesis. Several mutants for actin-related functions and lipid metabolic processes were specific to La (C6–C7).

The second pattern corresponds to the C2 and C11 clusters. Eleven mutants (2%, C2), which are related to the ubiquitin-dependent protein catabolic process *via* the multivesicular body sorting pathway (ESCRT complex), were specifically resistant to Ni, Y, and both Ln but sensitive to most of the other metals. Most of the C11 cluster mutants were also resistant to Ni, Y, and Yb but sensitive to La and were related to the retromer complex.

The third pattern included 139 mutants (22%, clusters C1, C9, and C10) sensitive to a range of metals, including REEs, whose functions are likely linked to the general metal stress response rather than Ln-specific, such as vesicular transport systems, as well as the vacuole and its acidification.

Last, a close relationship between Ln and Y emerged. Most of the mutants we identified (46%) were also either resistant or sensitive to Y (Figure 2). Y and Yb clustered closer than the two Ln together, which could be due to the close chemical properties of Y and Yb and therefore explain common cellular functions. The identification of many specific mutants in the Yb, La, or both screens underscores the unique cellular impact of Ln over other well-known metals.

### Ln Responses Differ From Well-Known Alkaline pH and Oxidative Stress-Mediated Responses

Oxidative stress is a widespread noxious consequence of exposure to some metals and metalloids, such as Cd and As, in yeast (Brennan and Schiestl, 1996; Litwin et al., 2013). Since similarities between Ln and As (32%) and between Ln- and Cd-responsive mutants (27%) were found (Figure 2), we further assessed whether Ln-mediated responses were common to oxidative stress-generating stressors in yeast. We compared our data with previous screens carried out on various chemical and physical stressors (Bennett et al., 2001; Chang et al., 2002; Aouida et al., 2004; Serrano et al., 2004; Thorpe et al., 2004) (Supplementary Figure S2; Supplementary Table S5). The highest similarity was found for both bleomycin and diamide but accounted for only 17% of the mutants responding to Ln and were mainly linked to genes involved in protein transport and transcription (Supplementary Figure S2). Although previous studies mentioned oxidative stress caused by Ln (Pagano et al., 2015), La and Yb clustered together as an outgroup, with more than 360 mutants specific to Ln, suggesting that the response to Ln stress is not primarily due to indirect stresses, such as pH or oxidative stress.

### The Golgi, ER, and Vacuole Are the Major Cellular Compartments Affected by Lanthanides

To highlight cellular hotspots of Ln responses, MIPS (Munich Information Center for Protein Sequences) subcellular localization analysis was performed using the identified mutants as a query (Supplementary Table S5). The disruption of genes linked to the vacuole and to the Golgi, which includes the Golgi membrane, Golgi to vacuole or to ER vesicle transport, led to cell sensitivity to both Ln, similar to Ni and Cd (Ruotolo et al., 2008), while some endosomal proteins were involved in Ln toxicity (resistant mutants). La-specific compartments were highlighted, with mutants for genes associated with the cytoplasm. Moreover, mutants for proteins of the vacuolar membrane and the nuclear envelope were sensitive to La, while impairing the actin cytoskeleton led to La resistance (Supplementary Table S6). Last, mutants for proteins of the ER membrane and the plasma membrane were specific for Yb. The lack of representation of the mitochondria was noticeable, which could be related to the low similarity between the Ln-mediated stress and chemical-induced oxidative stress.

### La and Yb Trigger Different Cellular Functions

**Figure 3:**
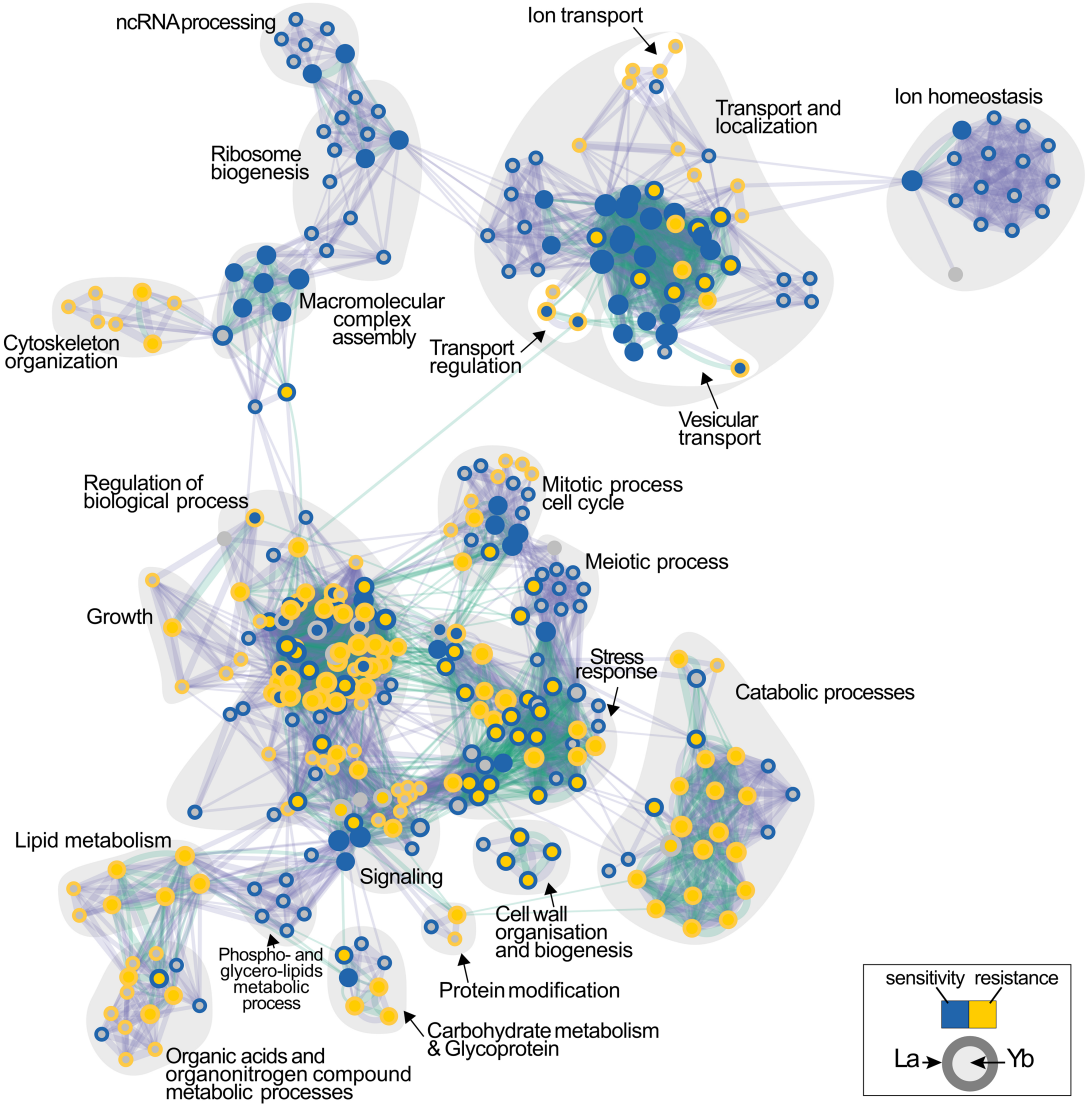
Functional enrichment analysis network of functions involved in the cellular response to lanthanides. Functions whose deletion renders cells either sensitive or resistant to La (outer circle) or Yb (inner circle) are shown in blue and yellow, respectively. Interlines represent gene overlap between two related functions, and edge width is proportional to the number of shared genes. The enrichment map was built using GSEA and visualized by the Enrichment map plug-in in Cytoscape.

To deepen the understanding of Ln-mediated toxicity mechanisms, the identification of the cellular processes engaged in Ln toxicity was evaluated through the analysis of biological pathways (Figure 3; Supplementary Table S7). Transport and localization functions, which include vacuolar transport, endocytosis and vesicle-mediated transport, catabolic processes, and lipid metabolism, were highly represented among La- and Yb-responsive mutants (Figure 3). Previously, endocytosis was also found as a primary pathway affected by these metals (Pallares et al., 2021) together with lipids, amino acids, and carbohydrates metabolisms (Grosjean et al., 2021).

However, relatively contrasting enrichment maps were generated for Yb and La (Figure 3), supporting that the two Ln involve diverse cellular functions in yeast. Functional categories, such as ion homeostasis, ribosome biogenesis, and ncRNA processing, were mainly specific to La. Other functions were less represented for Yb than for La, including mitotic and meiotic processes, together with metabolic processes involving organic acids and organo-nitrogen compounds. Several functions when altered caused sensitivity to La but resistance to Yb, such as cell wall organization and biogenesis and stress response pathways (Figure 3). Only a few functions enriched were mostly Yb-specific, such as signaling pathways, stress response, and catabolic processes (Supplementary Table S7). A wide range of mutants for proteins involved in translation were strictly sensitive to La, illustrated by cells lacking subunits of the ribosomal 60S and 40S subunit proteins (RPS and RPL proteins) (Figure 3; Supplementary Table S1). Given that many more mutants are sensitive to La (Figure 1), more La-responsive proteins could be required under La stress compared to Yb. Therefore, a reduced translation efficiency would be more detrimental under La stress.

This analysis allowed us to assign mutants to major functions involved in the response to Ln. This highlighted the components of the cell responses to La and Yb stress that belong to (i) the core component responsive to general Ln stress and (ii) the specific pathways involved in either La or Yb stress modulation. To further deepen our knowledge on the dissimilar response of yeast to La and Yb, we next focused our investigation on the role played by the cell wall and related signaling pathway in response to Ln.

### La and Yb Differentially Affect the Cell Wall and Related Signaling Pathway

**Figure 4:**
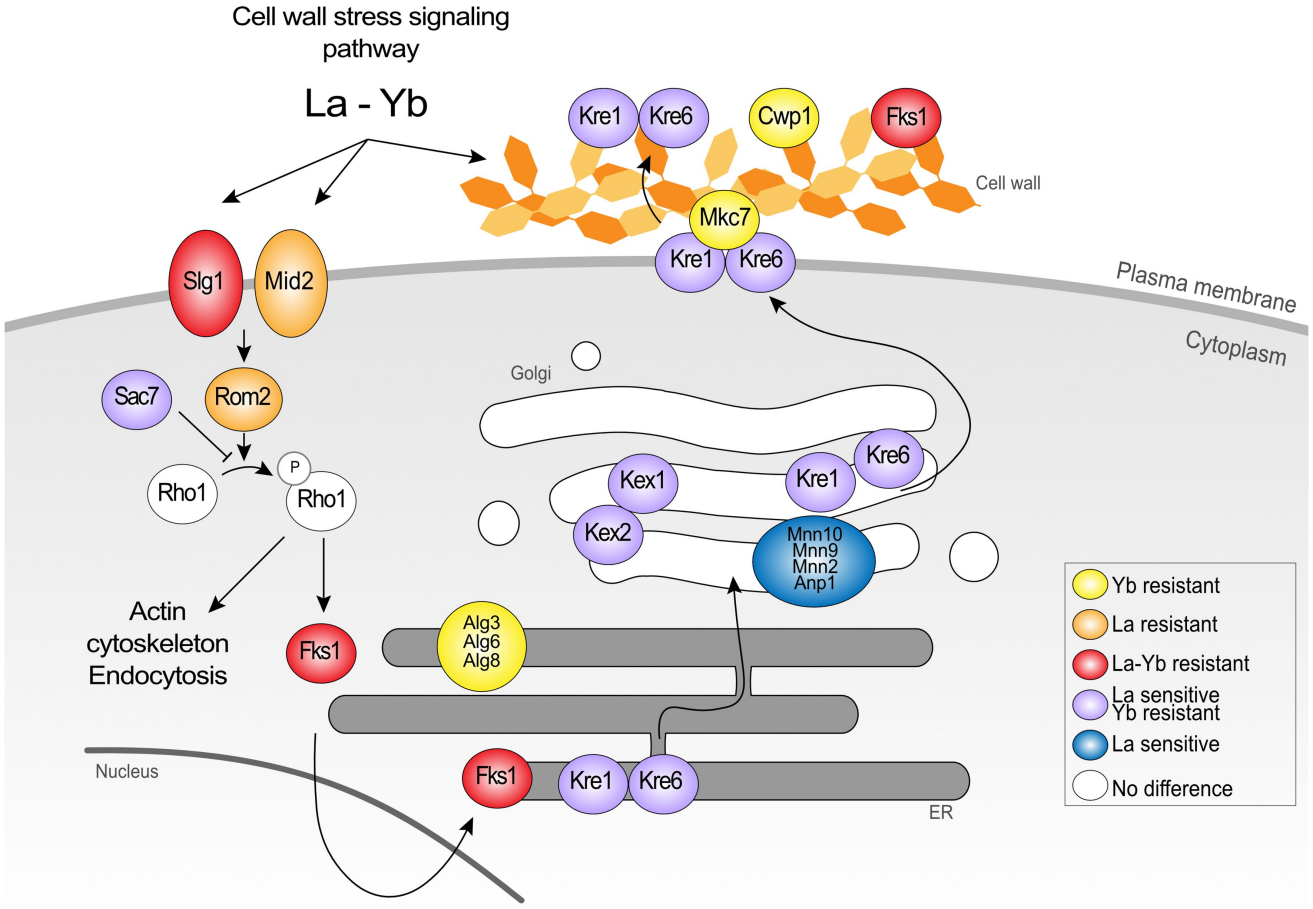
Schematic representation of the proteins belonging to the cell wall signaling pathway and cell wall organization in the response to Ln. The data show the dual involvement of the cell wall and signalization in the response to lanthanides.

The cell wall is the first shield against extracellular stressors, including metals. Several mutants responding to Ln exposure were knocked out for genes encoding proteins involved in the cell wall stress response and cell wall biosynthesis (Figures 3, 4; Supplementary Table S7). In a recent transcriptomics and proteomics analysis of *S. cerevisiae*, cell wall biosynthesis and organization-related pathways were upregulated in response to both La and Yb, supporting the strong implication of the cell wall as the first interface (Grosjean et al., 2021). However, the mutants found in the present deletome analysis displayed distinct phenotypes under La or Yb exposure, supporting the need of complementary multi-scale approaches. Mutants for mannosyltransferases (*mnn10*∆, *mnn9*∆, *mnn2*∆, and *anp1*∆) involved in cell wall mannoprotein synthesis were specifically sensitive to La, whereas mutants for glucosyltransferases involved in glycoprotein synthesis (*alg3*∆, *alg6*∆, and *alg8*∆) were specifically resistant to Yb (Figure 4; Supplementary Table S1).

**Figure 5:**
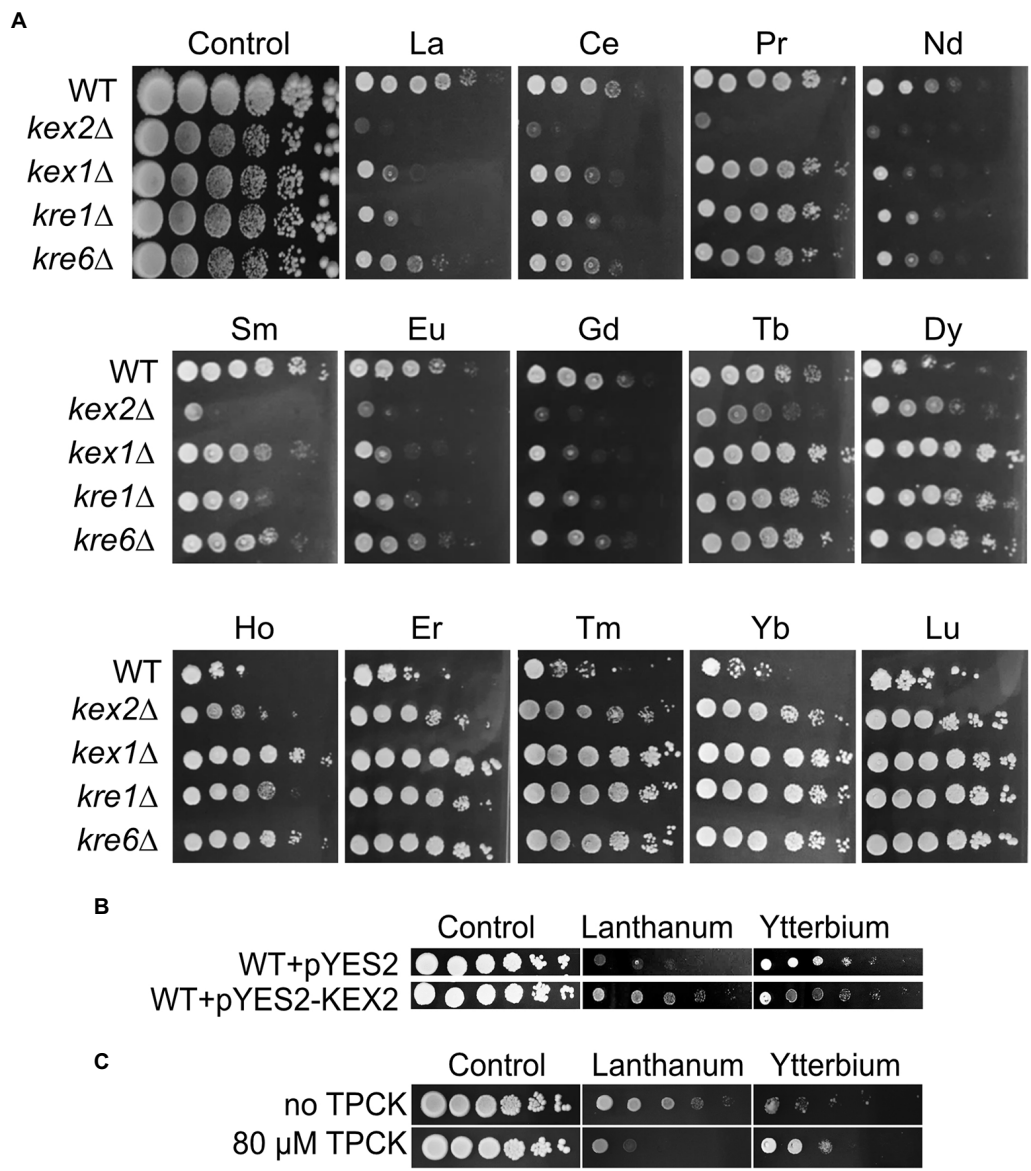
Growth of Kex and Kre mutants and effects of Kex2p overexpression or TPCK supplementation on yeast growth under Ln exposure. **(A)** Yeast growth was assessed on YPD medium without lanthanides (control) or supplemented with 4.0 mM La, 3.8 mM Ce, 5.8 mM Pr, 4.2 mM Nd, 3.9 mM Sm, 3.9 mM Eu, 3.8 mM Gd, 3.8 mM Tb, 3.6 mM Dy, 3.9 mM Ho, 3.9 mM Er, 3.6 mM Tm, 3.6 mM Yb, or 3.6 mM Lu, with 10-fold serial dilutions of cultures from left to right in each panel. A representative plate (out of 3 independent experiments) is shown. Plates were incubated for 5 days at 28°C. **(B)** Overexpression of Kex2p confers resistance to La but sensitivity to Yb. Yeast cells were transformed with the empty plasmid (pYES2) or with the same plasmid harboring KEX2 (pYES2-KEX2). Cells were grown on YPG in the presence of either La (3.8 mM) or Yb (3.2 mM) or in the absence of Ln (control), and 10-fold serial dilutions were plated on YPD media. **(C)** Drop test of 3-fold serial dilutions of the WT strain exposed to La (3.8 mM) and Yb (3.2 mM) with the addition of the serine protease inhibitor tosyl phenylalanyl chloromethyl ketone (TPCK). Representative pictures (out of 3 independent experiments) are given.

Remarkably, most of the cell wall-related mutants were resistant to Yb but sensitive to La, such as mutants in cell wall integrity-related proteins Kre1p, Kre6p, and Gas1p (Figure 4, 5; Supplementary Table S1). Mutants *kex2*∆ and *kex1*∆ also displayed this contrasted phenotype (Figures 4, 5), and Kex2p overexpression in WT cells reversed the phenotype (Figure 5B). Kex2p and Kex1p are both Ca-dependent serine proteases involved in the activation of secretory pathway and cell wall proteins (Figure 4), such as Scw4p (Fuller et al., 1989; Lesage and Bussey, 2006; Grbavac et al., 2017). Accordingly, exposure of WT yeast to the serine protease inhibitor tosyl phenylalanyl chloromethyl ketone (TPCK) resulted in phenotypes similar to those obtained with *kex2*∆ and *kex1*∆ (Figure 5C). We also found that Mkc7p, an aspartyl protease and member of the yapsin family acting downstream of Kex2p (Grbavac et al., 2017), was resistant to Yb only (Figure 4), supporting the importance of cell wall maintenance under Ln stress.

### Cell Wall Modifications Differentially Influence Ln Accumulation

**Figure 6:**
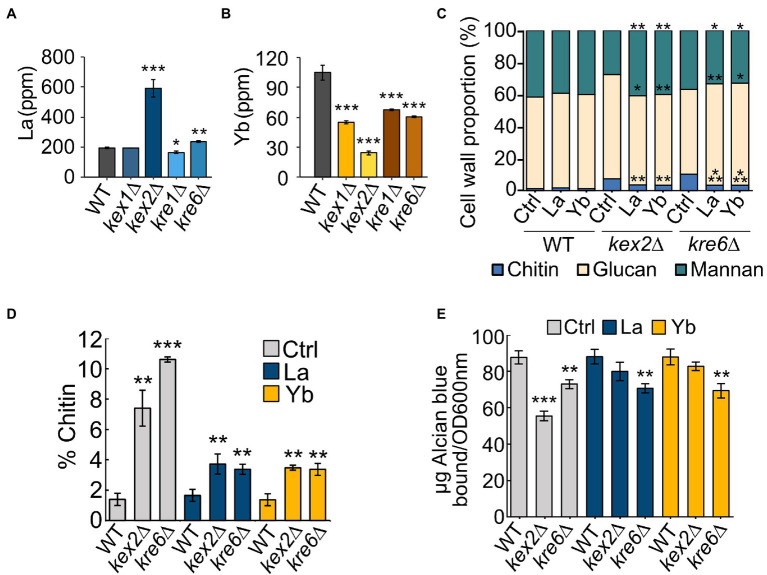
La and Yb concentrations and cell wall composition of WT and mutant strains. **(A,B)** Cells were grown in modified YNB and exposed to **(A)** La (50 μM) or **(B)** Yb (6 μM) for one hour. Data are the means (±SD) of three independent cultures. Significant differences from the wild-type condition are indicated by asterisks (ANOVA, Tukey HSD). **(C)** Cell wall composition of WT, kex2∆, and kre6∆ in chitin, glucans, and mannans. Significant differences from control conditions in each strain are indicated by asterisks (t-test). **(D)** Chitin content in the cell wall of WT, kex2∆, and kre6∆ under Ln exposure. Significant differences from the WT strain are indicated by asterisks (t-test). **(E)** Phosphomannan content using the Alcian blue binding test in each strain. Significant differences from the wild-type strain are indicated by asterisks (t-test). Data are the means (±SD) of three independent cultures. For all experiments: ^*^<0.05, ^**^<0.01, ^***^<0.001.

We assessed whether cell wall integrity components had a role in different Ln accumulation by measuring La and Yb concentrations in WT and mutant cells (Figures 6A,B). Interestingly, *kre1*∆, *kre6*∆, *kex1*∆, and *kex2*∆ accumulated two to three times less Yb (Figure 6B) than the WT, correlating with their resistance to Yb. Conversely, *kex2*∆ and *kre6*∆ accumulated significantly more La (Figure 6A), concordant with their higher sensitivity to La (Figure 5A). Consequently, these proteins and related cell wall components could be involved in (i) Ln differential adsorption followed by an internalization step or (ii) by affecting Ln uptake through the modulation of ion membrane transporters.

### La and Yb Exposure Modify Cell Wall Structure and Composition

To investigate whether cell wall composition modulates Ln interactions, the cell wall composition of WT, *kex2*∆, and *kre6*∆ was analyzed under Ln exposure (Figures 6C–E; Supplementary Figure S3). The chitin content of WT cells remained constant despite Ln exposure. Interestingly, upregulation of the chitin cell wall metabolic process and the 1,3-β-D-glucan biosynthesis pathways were observed by transcriptomics and proteomics (Grosjean et al., 2021). This suggests that upregulation of these cell wall-related pathways is essential to maintain a proper cell wall structure, composition, and organization to compensate for Ln binding. Relatively negligible differences in β-1,3-glucan and cell wall proteins were observed between the different conditions and strains (Supplementary Figures S3B,D). However, independent of the absence or presence of Ln, the chitin content at least doubled in both *kex2*∆ and *kre6*∆ relatively to WT, which supports that these cell wall modifications are a consequence of these mutations (Figures 6C,D; Supplementary Figure S3A). Additionally, while the amount of mannans remained relatively unchanged, lower amounts of phosphomannans and O-linked mannans were observed in the mutants (Figure 6E; Supplementary Figures S3C,E,F). A previous study demonstrated the adsorption of Yb and subsequent formation of Yb-phosphate crystals onto the cell wall of *S. cerevisiae* (Jiang et al., 2012). Other studies mentioned the adsorption of Ln on extracellular biopolymers, such as the cell wall of certain bacteria (Moriwaki and Yamamoto, 2013). It was further argued that LREEs (La) preferentially bind to phosphate groups, while HREEs (Yb) equally bind to phosphate and carboxyl groups (Ngwenya et al., 2009). Consequently, the relative proportions of chitin and phosphomannan could modulate the proportion of functional groups to which Ln could differentially bind. The lower abundance of phosphate groups containing phosphomannans could reduce La adsorption, therefore increasing the free La accumulation potential and toxicity (Figure 6A). Conversely, reduced phosphomannans combined with higher chitin content would increase the proportion of carboxyl groups, allowing greater Yb adsorption synonymous to an increased Yb resistance. To confirm the hypothesis of a selective LREE and HREE binding potential, we evaluated the growth of *kex2*∆, *kex1*∆, *kre1*∆, and *kre6*∆ with 14 different Ln (Figure 5A). We observed that *kex2*∆ and, to a lesser extent, *kex1*∆, *kre1*∆, and *kre6*∆ were all sensitive to LREEs (La to Eu), while being gradually resistant with heavier Ln. These observations support the differential interaction of LREEs and HREEs with the cell wall based on their chemical properties.

### Cell Wall Modifications Disturb Protein Abundances

**Figure 7:**
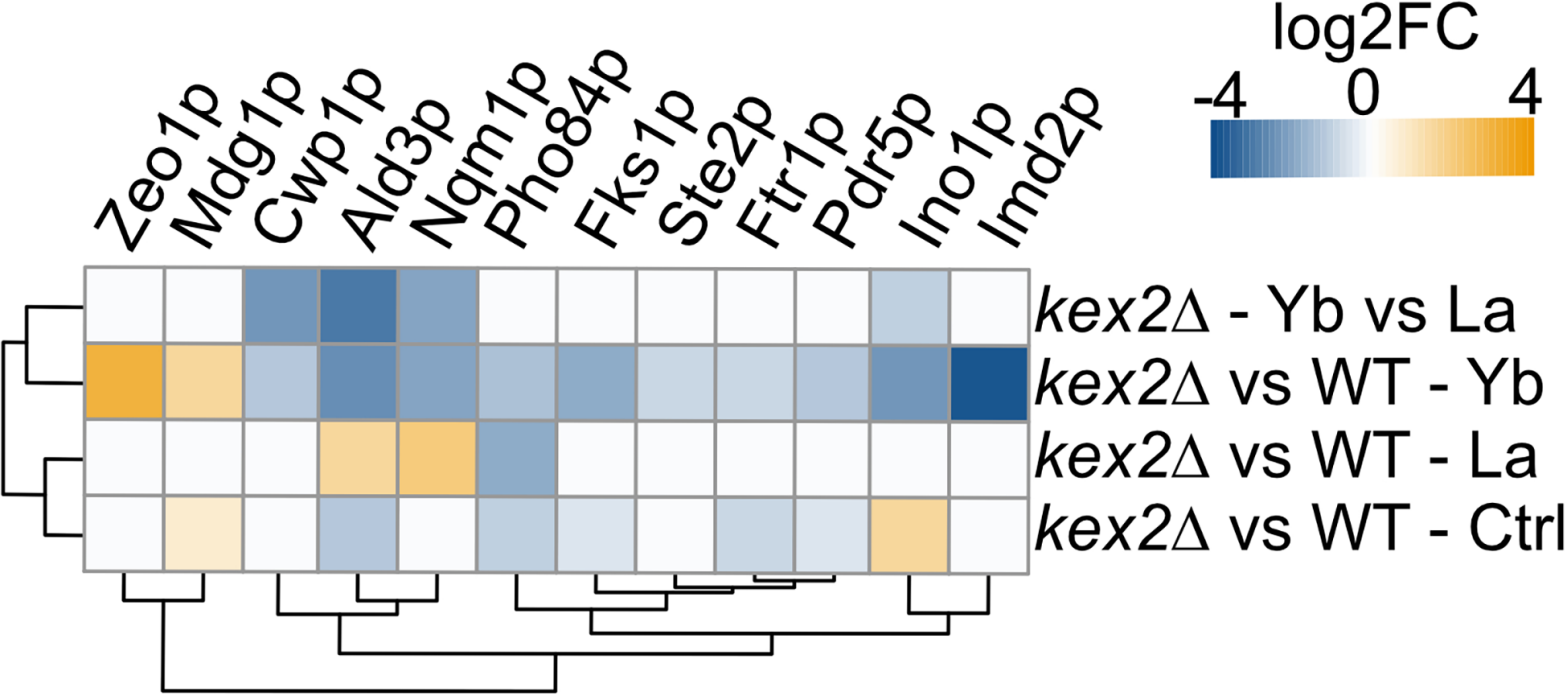
Heatmap displaying protein abundance changes in kex2∆ compared to the WT. Please, refer to the Supplementary text for information related to proteomics data acquisition and analysis.

Since *kex2*∆ displayed the most contrasting phenotype, we leveraged label-free high-throughput shotgun proteomics to identify proteome modulations under Ln exposure (Figure 7; Supplementary Figure S4; Supplementary Table S8). Seventy-five proteins displayed different abundances between La and Yb in *kex2*∆, and twelve interesting candidates, because of their biological role, are shown in Figure 7. Nqm1p and Ald3p were both repressed under Yb but more abundant under La. Nqm1p is a transaldolase upregulated by various stresses, including osmotic shock (Michel et al., 2015), while Ald3p is a stress-responsive aldehyde dehydrogenase (Navarro-Aviño et al., 1999). Upregulation of these two proteins under La could be the result of osmotic stress induced by important La absorption in *kex2*∆ (Figure 6A). Interestingly, several membrane transporters had a lower abundance in *kex2*∆ than in WT. Pdr5p, a plasma membrane ABC multidrug transporter involved in cation resistance (Miyaharan et al., 1996), was specifically repressed under Yb (Figure 7). Similarly, Pho84p, a high-affinity phosphate transporter and low-affinity Mn transporter, and Ftr1p, a high-affinity iron transporter (Figure 7), were less abundant in *kex2*∆. We can hypothesize that Yb could be taken up through these transporters, which would explain the higher resistance of *kex2*∆ to Yb by decreasing its putative non-specific uptake.

### La and Yb Trigger the Cell Wall-Related Signaling Pathway

This genome-wide deletion mutant screen further highlighted mutants for genes related to signaling pathway (Figure 4). Again, a segregation between La and Yb was observed. The cell wall stress signaling pathway was represented notably by the plasma membrane receptor *mid2*∆ and the GTP exchange factor *rom2*∆, both of which are resistant to La (Figure 4). Additionally, Zeo1p, which acts antagonistically to Mid2p, was significantly more abundant in *kex2*∆ (Figure 7). *slg1*∆, a cell wall integrity sensor (Mao et al., 2011), and *fks1*∆ were also resistant to both Ln. Furthermore, *sac7*∆, a negative regulator of the RHO1-PKC1-MAPK cell integrity pathway, displayed the same phenotype as *kre*∆ and *kex*∆, suggesting the dual role of the cell wall and its ability to respond to different Ln (Figure 4). Altogether, these results suggest that the cell wall not only acts as a Ln sequestration/adsorption compartment but is also an active signal effector differentiating LREEs and HREEs.

## Conclusion

**Figure 8:**
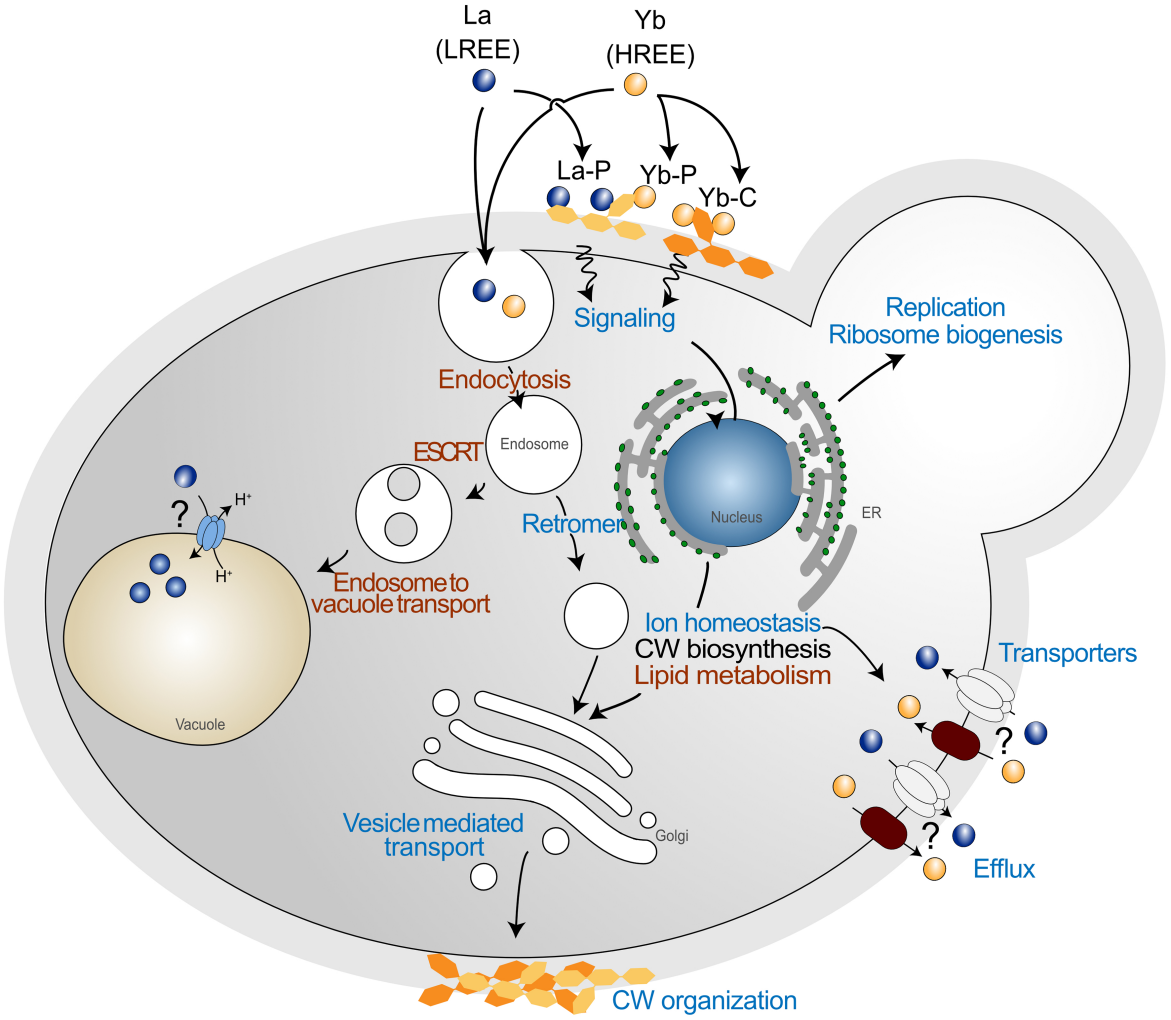
Global pathways in *Saccharomyces cerevisiae* exposed to lanthanide stress. Schematic representation of general functions identified through mutant collection screening. Common functions between the two lanthanides are shown in red, and functions showing a different behavior are shown in blue. La is representative of LREEs, while Yb is representative of HREEs. La-P and Yb-P represent lanthanum ions bound to phosphate groups, while Yb-C represents ytterbium ions bound to carboxyl groups.

To date, studies dealing with Ln toxicity are scarce, and how organisms can cope with the toxicity of these emerging contaminants remains an open question. In the present study, we adopted a well-defined metal toxicogenomics analysis in which yeast were subjected to a high Ln-mediated stress comparatively to a recent study (Pallares et al., 2021). Interestingly, we confirmed in our study that the vesicle transport and endocytosis pathways were strongly affected by Ln. However, we report several other pathways that were also impacted by a high Ln stress, including lipid metabolism, ion homeostasis, cell wall organization, and related signaling pathway (Figure 8), which were not reported previously (Pallares et al., 2021), extending our knowledge on the cellular and molecular responses of yeast exposed to Ln. Additionally, by using an arrayed mutant collection screening, we demonstrated that some mutants that were previously found specific to HREEs, are also shared with La (e.g., ypl056c∆, ykr020w∆, ypl057c∆, ybr036c∆), expending the yeast core response to Ln, but also supporting the need for combining experimental approaches. In a recent paper, we combined transcriptomics and proteomics analyses on *Saccharomyces cerevisiae* exposed to La and Yb and most of the pathways impacted by Ln were found in each of these complementary approaches (Grosjean et al., 2021). This multi-scale analysis (deletome, transcriptome, and proteome) of the response of yeast to Ln provides valuable target genes, proteins, and pathways involved in Ln toxicity response and detoxification that complements and expands on previous studies.

Moreover, we highlighted that the response pathways were modulated differently depending on the nature of the different Ln. The high reactivity of Ln to functional groups of the cell wall makes this compartment the first layer discriminating different Ln. The cell wall stress-related signaling pathway might induce the modification of cell wall organization as well as the lipid composition of membranes in response to Ln (Figure 8). Such a close relationship between membrane lipids and adaptation to environmental stress has been shown to impact Ca influx channels (Jiang et al., 2019), by which Ln could be taken up (Ene et al., 2015). Additionally, Ln activate intracellular signaling, accumulation of phosphatidylinositol-4-phosphate at the plasma membrane, and cytoskeleton-dependent rearrangement complexes in Arabidopsis (Lee et al., 2020). Therefore, a different mechanism to regulate Ln membrane transporters should also be considered, such as through endocytosis (Figure 8). The recurrence of Mn, Zn, and Fe transporters in our deletomic and proteomic analyses are in concordance with our previous whole cell transcriptomics and proteomics analysis on wild-type *S. cerevisiae*, for which we observed inhibition of gene expression and reduced protein abundance of these transporters (e.g., Ftr1-Fet3, Zrt1, Smf1; Grosjean et al., 2021). The identification of these cation transporters by several multi-scale and complementary approaches also calls for further investigations on these uptake pathways as a way of Ln entry in cells and their involvement in the disruption of micronutrient homeostasis.

Given the differences observed between LREEs and HREEs, engineering yeast cells to optimize REE binding (modified cell wall) and/or accumulation deserves further attention. Yeasts could be used as a biotechnology tool for REE purification in mixed metal or Ln solutions (Sun et al., 2019). Finally, in their environment, organisms including humans may be exposed to a single REE species or to a mixture of LREEs and HREEs. Considering the dissimilarities observed between La and Yb in our study, exposure to mixed Ln could display additive or antagonistic effects (Morel et al., 2021), which would require further investigation.

These results on *S. cerevisiae* are a first step in the delineation of Ln toxicity mechanisms and will enable future investigations on more complex eukaryotes, to improve risk assessment toward Ln. The high number of yeast Ln-responsive mutants for genes holding human counterparts sets a promising basis for further studies on the impact of Ln toxicity on human health.

## Data Availability Statement

The datasets presented in this study can be found in online repositories. The names of the repository/repositories and accession number(s) can be found in the article/Supplementary Material.

## Author Contributions

NG: investigation, conceptualization, formal analysis, writing—original draft, and writing—review and editing. MLJ: conceptualization, formal analysis, supervision, and writing—review and editing. MC, HM-M, and JA: investigation, formal analysis, and writing—review and editing. EG: supervision and writing—review and editing. DB: investigation, conceptualization, formal analysis, supervision, project funding, and writing—review and editing. All authors contributed to the article and approved the submitted version.

## Funding

This work was supported by the French National Research Agency through the National Program “Investissements d’Avenir” with the reference ANR-10-LABX-21-01/LABEX RESSOURCES 21 and by the Region Grand Est. HM-M is supported by Consejo Nacional de Ciencia y Tecnología, México (ref. *CF*-2019-6380).

## Conflict of Interest

The authors declare that the research was conducted in the absence of any commercial or financial relationships that could be construed as a potential conflict of interest.

## Publisher’s Note

All claims expressed in this article are solely those of the authors and do not necessarily represent those of their affiliated organizations, or those of the publisher, the editors and the reviewers. Any product that may be evaluated in this article, or claim that may be made by its manufacturer, is not guaranteed or endorsed by the publisher.

## Footnotes

^1^ http://yeastmine.yeastgenome.org/yeastmine/

^2^ http://funspec.med.utoronto.ca

^3^ http://bioinformatics.org/go2msig/
