# Supplementary material for: Genome-Wide Mutant Screening in Yeast Reveals that the Cell Wall is a First Shield to Discriminate Light From Heavy Lanthanides
Source: Front Microbiol. 2022 May 19;13:881535. doi: 10.3389/fmicb.2022.881535 (PMC9162579; doi:10.3389/fmicb.2022.881535)
Supplement: Supplementary file 1 [file Data_Sheet_1.docx]

# Supplementary text and figures

Genome-wide mutant screening in yeast reveals that the cell wall is a first shield to discriminate light from heavy lanthanides

Nicolas Grosjean^1,2,†^, Marie Le Jean^2^, Michel Chalot^3,4^, Héctor M. Mora-Montes^5^, Jean Armengaud^6^, Elisabeth M. Gross^2^, Damien Blaudez^1^*

*^1^Université de Lorraine, CNRS, LIEC, F-54000 Nancy, France*

*^2^Université de Lorraine, CNRS, LIEC, F-57000 Metz, France*

*^3^Université de Bourgogne Franche-Comté, CNRS, Laboratoire Chrono-Environnement, F-25000 Besançon, France*

*^4^Université de Lorraine, F-54000 Nancy, France*

*^5^Departamento de Biología, División de Ciencias Naturales y Exactas, Campus Guanajuato, Universidad de Guanajuato, Noria Alta s/n, col. Noria Alta, C.P., Guanajuato Gto. 36050, Mexico*

*^6^Université Paris-Saclay, CEA, INRAE, Département Médicaments et Technologies pour la Santé (DMTS), SPI, F-30200 Bagnols-sur-Cèze, France*

*†Current affiliation; Biology Department, Brookhaven National Laboratory, Upton, NY 11973, USA*

*Corresponding author:

Dr Damien Blaudez

UMR 7360 LIEC, Faculté des Sciences et Technologies

Université de Lorraine, BP70239, Vandoeuvre-lès-Nancy F-54506, France

Tel: +33-3-72-74-51-67, E-mail: [damien.blaudez@univ-lorraine.fr](mailto:damien.blaudez@univ-lorraine.fr)

**7 pages, supplementary text, 4 Figures (S1-S4)**

**Supplementary text (Experimental procedures)**

**Sample preparation for proteomics analysis**

Yeast was grown as mentioned above. Cultures were inoculated at an OD_600nm_ of 0.05, and once the cultures reached an OD_600nm_ of 0.6, REEs were added at the EC_50_ and exposed for 1 or 4 h. Cells were harvested at room temperature at 4000 rpm for 1 min. Cell pellets were washed twice with PBS 50 mM (pH 7) and snap frozen. Proteins were extracted from frozen cells by bead beating with a Precellys 24 instrument (Bertin) after the addition of LDS1X (Invitrogen) consisting of 106 mM Tris/HCl, 141 mM Tris base, 2 % lithium dodecyl sulfate, 10 % glycerol, 0.51 mM EDTA, 0.22 mM SERVA Blue G250, 0.175 mM phenol red, buffered at pH 8.5 and supplemented with 2.5 % ß-mercaptoethanol. Samples were heated for 5 min at 95 °C, loaded onto a denaturing 4–12 % gradient 10-well NuPAGE (Invitrogen) gel (25 µL per well), and then subjected to electrophoresis for 5 min as previously recommended 2. After quick staining with Coomassie Blue Safe (Invitrogen), polyacrylamide bands corresponding to the whole proteomes were sliced, destained with ultrapure water, reduced with dithiothreitol, and treated with iodoacetamide before performing proteolysis with Trypsin Gold Mass Spectrometry Grade protease (Promega) in the presence of 0.01 % ProteaseMAX surfactant (Promega).

**Tandem mass spectrometry**

Tryptic peptides were subjected to tandem mass spectrometry with a Q-Exactive HF tandem mass spectrometer (Thermo Fisher Scientific) incorporating an ultra-high field Orbitrap analyser coupled in line with an Ultimate 3000 chromatography system (Thermo Fisher Scientific). The tandem mass spectrometer was operated in the data-dependent mode as described ^3^. Briefly, each sample (4 µL) was injected and desalted on a reverse-phase capillary C18 PepMapTM 100 precolumn (Thermo Fisher Scientific), and then peptides were resolved based on their hydrophobicity on a nanoscale 500-mm C18 PepMapTM 100 (5 mm x 300 µm I.D., Thermo Fisher Scientific) column using a 90-min gradient from 2.5 % to 40 % of 80 % CH_3_CN, 20 % H_2_O, and 0.1 % formic acid. Once separated, mass spectra for peptides were acquired in the full-scan range 350-1800 *m/z* at a resolution of 60,000 and an AGC target of 3.10^6^. Peptide ions with charges ^2+^ or ^3+^ were selected for fragmentation according to the Top20 method. MS/MS mass spectra were acquired with an AGC target set at 10^4^, a loop count of 60 ms, an isolation window of 1.6 *m/z*, a resolution of 15,000, a dynamic exclusion of 20 s, and a scan performed over a dynamic mass range from the first mass detected up to 15 times the first mass.

**Peptide-to-spectrum assignment and protein identification.**

Peak lists were automatically generated from raw files with an in-house script using the Proteome Discoverer 1.4.1 (Thermo Fisher Scientific) daemon conversion function and the following options: minimum mass (400), maximum mass (5,000), grouping tolerance (0), intermediate scans (0), and threshold (1,000). MS/MS spectra from the resulting *.mgf files were assigned to *S. cerevisiae* peptide sequences with Mascot software v2.5.1 (Matrix Science) with the following parameters: full trypsin specificity with up to two missed cleavages allowed, static modification of carbamidomethylated cysteine, variable deamidation of asparagine and glutamine, variable oxidation of methionine, mass tolerance of 5 ppm on parent ions and mass tolerance on MS/MS of 0.02 Da. Peptide matches with a MASCOT peptide score below a p-value of 0.05 were parsed with IRMA software ^4^. Proteins were validated when at least two different peptides were detected. The false discovery rate for protein identification was below 1 %, as estimated with the reverse database decoy MASCOT search option. Spectral counts corresponding to the number of MS/MS spectra assigned per protein were extracted for each sample without applying the parsimony rule to avoid isoform quantitation bias. The normalized spectral abundance factor (NSAF) for each protein was calculated as the total spectral count divided by its molecular mass in kDa and presented as a percentage. Spectral counts were compared between conditions using the T-Fold method of PatternLab software ^5^ after normalizing spectral counts. The statistical classes were defined as “Blue” for proteins that satisfied both the |T-fold| (≥ 1.5) and its associated p-value (≤ 0.05), “Orange” for proteins with identifications that did not meet the T-fold criterion (but they deserve a second look because they had low p-values), “Green” for proteins that satisfied the fold criteria but were most likely false positives, and “Red” for proteins that did not meet the fold and p-value criteria.

**References**

(1) Okada, H.; Abe, M.; Asakawa-Minemura, M.; Hirata, A.; Qadota, H.; Morishita, K.; Ohnuki, S.; Nogami, S.; Ohya, Y. Multiple Functional Domains of the Yeast l,3-β-Glucan Synthase Subunit Fks1p Revealed by Quantitative Phenotypic Analysis of Temperature-Sensitive Mutants. *Genetics* **2010**, *184* (4), 1013–1024. https://doi.org/10.1534/genetics.109.109892.

(2) Hartmann, E. M.; Allain, F.; Gaillard, J.; Pible, O.; Armengaud, J. Taking the Shortcut for High-Throughput Shotgun Proteomic Analysis of Bacteria. In *Host-Bacteria Interactions. Methods in Molecular Biology (Methods and Protocols)*; Vergunst, A., O’Callaghan, D., Eds.; Humana Press: New York, NY, 2014; Vol. 1197. https://doi.org/10.1007/978-1-4939-1261-2.

(3) Klein, G.; Mathé, C.; Biola-Clier, M.; Devineau, S.; Drouineau, E.; Hatem, E.; Marichal, L.; Alonso, B.; Gaillard, J. C.; Lagniel, G.; Armengaud, J.; Carrière, M.; Chédin, S.; Boulard, Y.; Pin, S.; Renault, J. P.; Aude, J. C.; Labarre, J. RNA-Binding Proteins Are a Major Target of Silica Nanoparticles in Cell Extracts. *Nanotoxicology* **2016**, *10* (10), 1555–1564. https://doi.org/10.1080/17435390.2016.1244299.

(4) Dupierris, V.; Masselon, C.; Court, M.; Kieffer-Jaquinod, S.; Bruley, C. A Toolbox for Validation of Mass Spectrometry Peptides Identification and Generation of Database: IRMa. *Bioinformatics* **2009**, *25* (15), 1980–1981. https://doi.org/10.1093/bioinformatics/btp301.

(5) Carvalho, P. C.; Yates, J. R.; Barbosa, V. C. Improving the TFold Test for Differential Shotgun Proteomics. *Bioinformatics* **2012**, *28* (12), 1652–1654. https://doi.org/10.1093/bioinformatics/bts247.

**
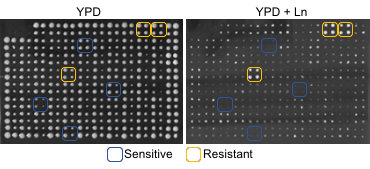
**

**Figure S1. Representative primary screens data for lanthanum- and ytterbium- responsive mutants.** Representative 384-well format growth tests for the lanthanum and ytterbium toxicity primary screens (four pin replication of each mutant). A control plate (YPD medium) and the same plates supplemented with 4.5 mM lanthanum or 3.8 mM ytterbium are shown. Putative lanthanide sensitive (blue) and resistant mutants (orange) are highlighted.


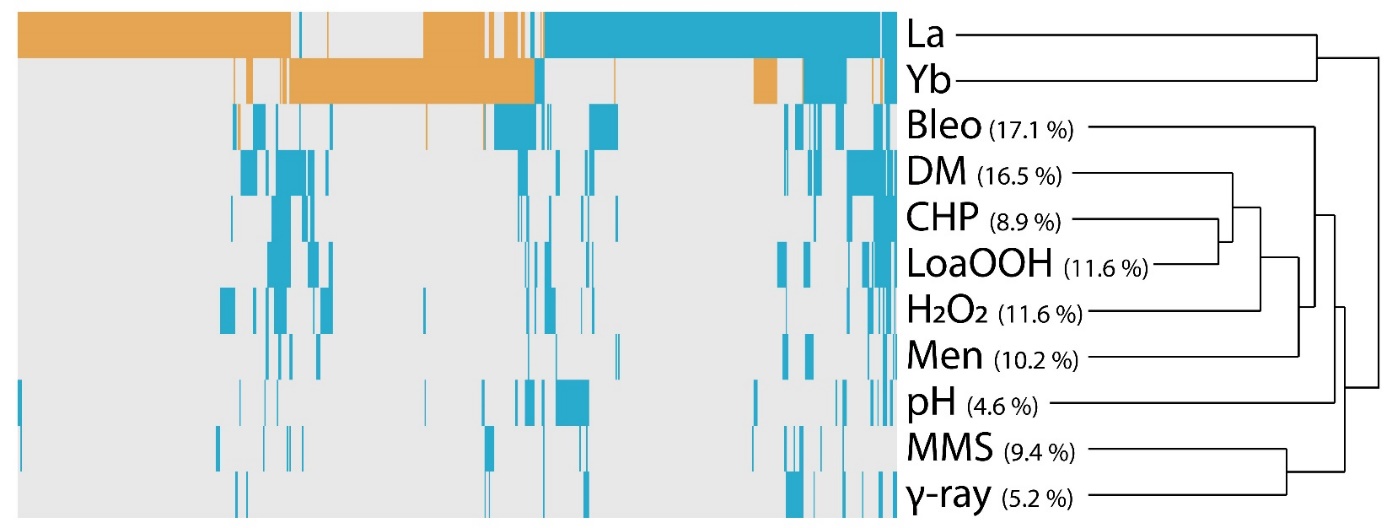


**Figure S2. Cross comparison of mutant phenotypes to lanthanides versus other nonmetallic stressors.** Hierarchical clustering of La and Yb sensitivity/tolerance-conferring mutations with the mutant sensitivity/tolerance profiles of other stressors. References to these works are provided in Supp Table S4. The x-axis corresponds to gene deletions and the y-axis indicates the different stressors from previous yeast genomic phenotyping screens conducted on deletion mutant collections; Non-metal stressors were selected: methyl methane sulfonate (MMS), γ-radiation (γ-ray), bleomycin (Bleo), alkaline pH (pH), menadione (Men), hydrogen peroxide (H_2_O_2_), cumene hydroperoxide (CHP), linoleic acid 13-hydroperoxide (LoaOOH), and diamide (DM). Only mutants displaying a phenotype on lanthanides were shown in this analysis. Mutant strains exhibiting either an enhanced sensitivity, a higher tolerance or no phenotype are shown in blue, orange and gray, respectively. Values into brackets after the element denote the percentage of mutants that were found common between the present screening (La and Yb) and screenings on other non-metallic stressors.


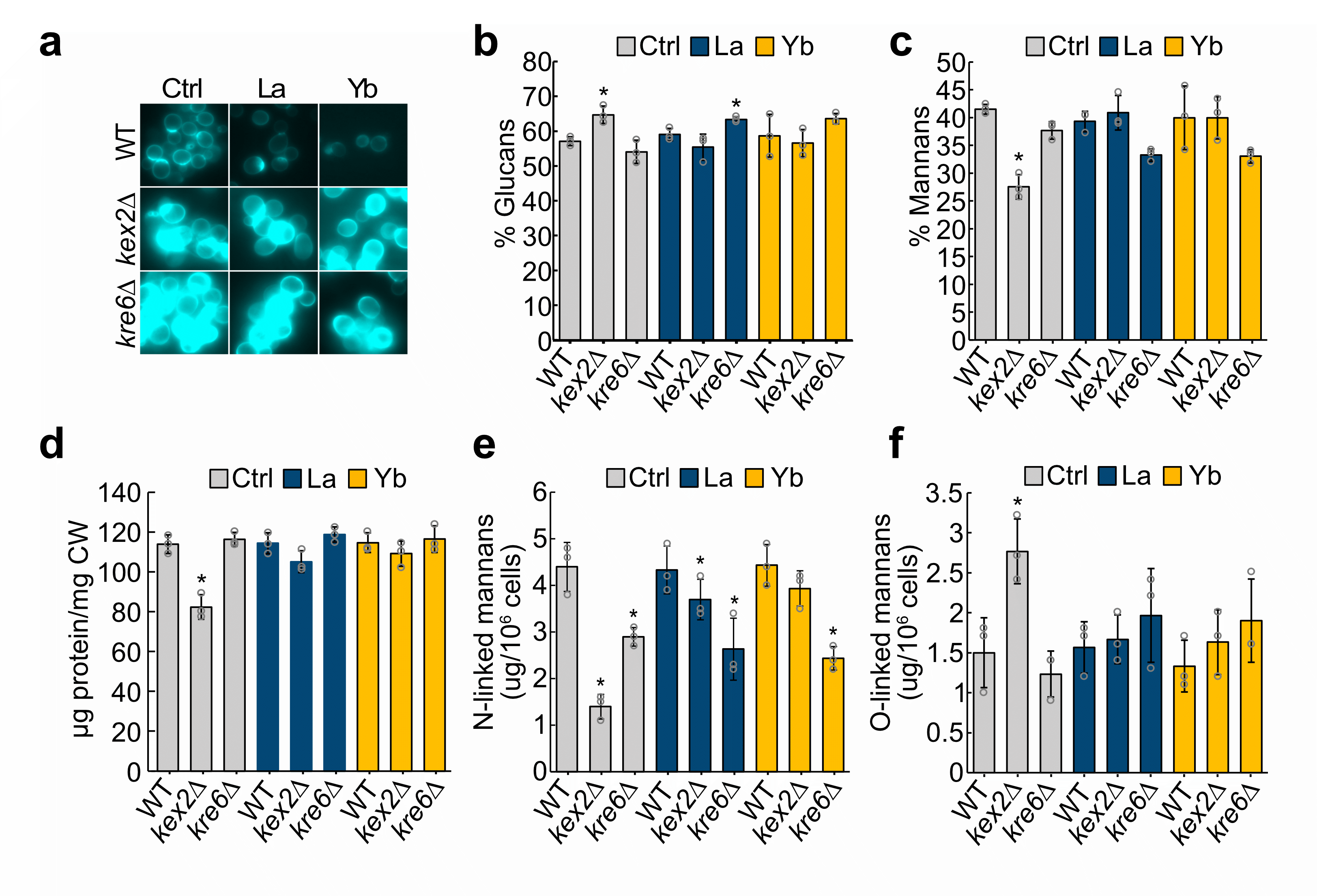


**Figure S3. Cell wall composition under Ln exposure.** Yeast cells were exposed to lanthanides and cell wall composition was analyzed. (a) Chitin staining using calcofluor white in WT, ∆Kex2, and ∆Kre6, under Ln exposure. Representative pictures are given. (b) Relative proportion of the cell wall in glucans. (c) Relative proportion of the cell wall in mannans. (d) Cell wall protein content. (e) N-linked mannan content. (f) O-linked mannan content.


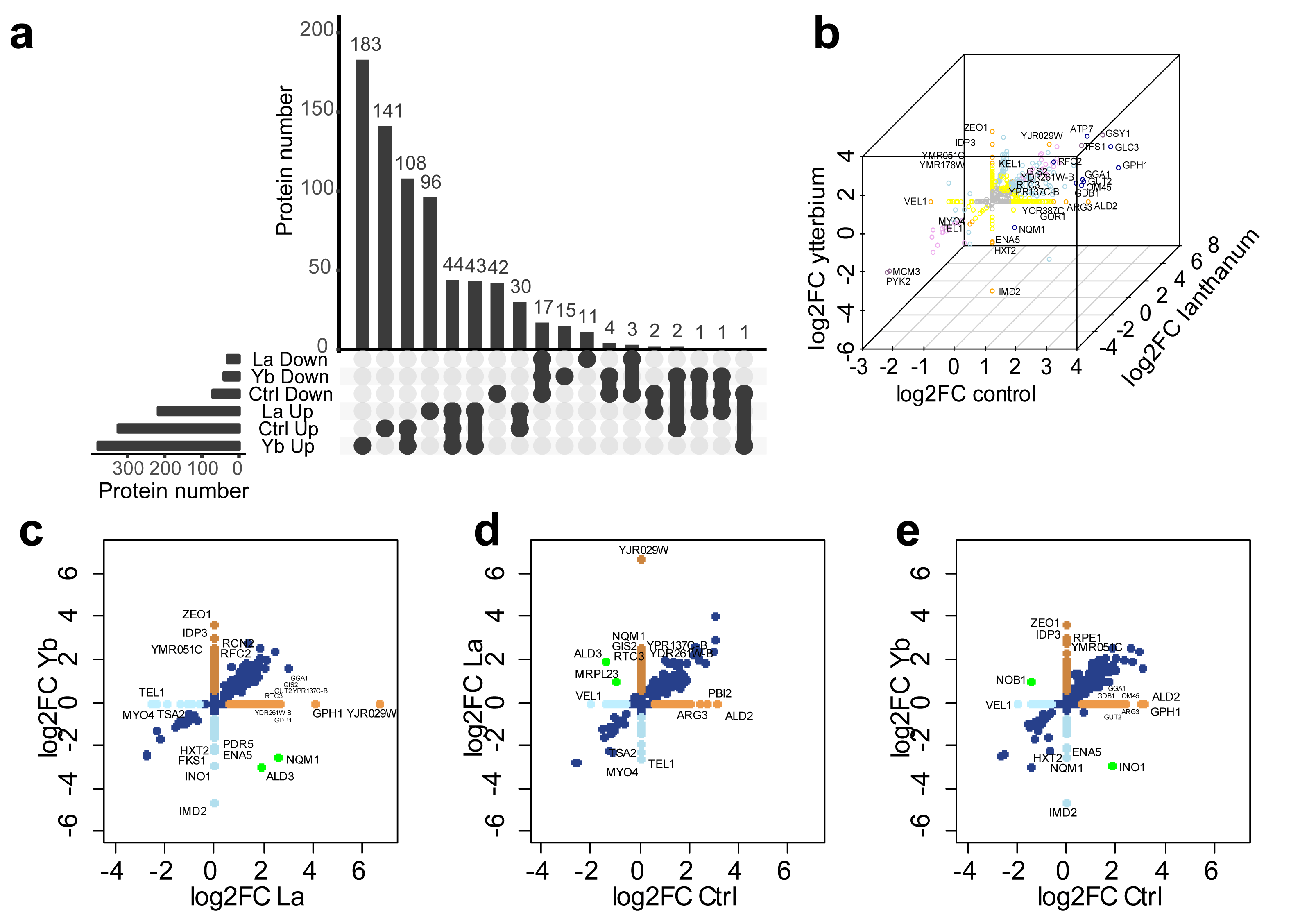


**Figure S4. Proteomic analysis of the *KEX2* mutant under control, lanthanum, and ytterbium exposure.** (a) UpSetR plot displaying the number of proteins up- and down-regulated shared between the three conditions tested. (b) 3D scatter plot of Log2 fold change (Log2FC) of proteins expressed in the mutant relatively to the WT, under control, lanthanum and ytterbium conditions. (c) Scatter plot of the Log2FC of proteins expressed in the mutant relatively to the WT, under lanthanum and ytterbium conditions. (d) Same as c, but comparing of lanthanum and control conditions. (e) Same as c, comparing ytterbium and control conditions. Green dots represent proteins whose expression is opposite in two conditions.
